# Expression, Purification, and Characterization of Cu/ZnSOD from *Panax* Ginseng

**DOI:** 10.3390/molecules19068112

**Published:** 2014-06-16

**Authors:** Dayong Ding, Shichao Liu, Kai Wang, Lihong Huang, Jisheng Zhao

**Affiliations:** 1 Department of Gastrointestinal Surgery, China-Japan Union Hospital, Jilin University, Changchun 130000, China; E-Mail: dyangel89@163.com; 2 Center of Chinese Medicine and Bio-Engineering Research, Changchun University of Chinese Medicine, Changchun 130117, China; 3 Department of Rheumatoid Immune, First Hospital of Jilin University, Changchun 130000, China; E-Mail: caikexin5258@126.com; 4 Department of Geriatrics, China-Japan Union Hospital, Jilin University, Changchun 130000, China; E-Mail: 18946726604@163.com

**Keywords:** ginseng, Cu/ZnSOD, pBV220, expression, purification

## Abstract

Superoxide dismutase (SOD) has a strong antioxidant effect, but the traditional SOD extraction method is not the most efficient method of SOD amplification. In this study, we report the cloning of the Cu/ZnSOD gene from *Panax* ginseng into a temperature-regulated expression plasmid, pBV220. Cu/ZnSOD inclusion bodies were expressed in *E. coli* at a high level. Then, the inclusion bodies were purified by ion-exchange chromatography and molecular sieve chromatography. Finally, we obtained stable SOD in the bacterial broth, with a protein content of 965 mg/L and enzyme specific activity of 9389.96 U/mg. These results provide a foundation for future studies on the antioxidant mechanisms of ginseng and the development and application of ginseng Cu/ZnSOD.

## 1. Introduction

*Panax* ginseng, a traditional Chinese medicinal herb, is well known for its variety of pharmacological actions, in particular its strong antioxidant effect [1]. Previous studies have shown that the SOD metalloenzyme can maintain the dynamic balance of the production and elimination of O_2_ in organisms [2,3,4,5,6] and may be an important factor in the antioxidant effects of ginseng [7,8,9,10]. We previously reported the extraction of Cu/ZnSOD from ginseng [11]; however, there were some disadvantages to that method of extraction. The yield was only 0.65%, so this extraction method required a large amount of fresh raw herbs. It is necessary to solve not only the problem of resource consumption associated with the extraction of Cu/ZnSOD, but also the complexity of the extraction process and the time limitation for specimen collection. All of these disadvantages hinder the expanded production of ginseng SOD. Fortunately, recombinant DNA technology can solve these problems and is suitable for industrial production.

IPTG (isopropyl β-D-thiogalactoside) is a strong inducer that is widely used in laboratories because of its stability. The disadvantages of using IPTG are its high cost and its toxicity. It can significantly inhibit colony growth and may even harm people if it is inhaled, ingested or absorbed through the skin. Therefore, a temperature-controlled expression vector was used instead of IPTG. The vector, pBV220, is non-poisonous, and its expression is induced simply by adjusting the temperature to approximately 42 °C [12]. 

For the purpose of improving the research and production of strong antioxidants, we cloned the Cu/ZnSOD genes from ginseng and successfully constructed the pBV220-Cu/ZnSOD expression vector. The vector efficiently expressed the Cu/ZnSOD genes in *E. coli* and generated a pure protein with high enzymatic activity. These results may help to elucidate the antioxidation mechanism of Cu/ZnSOD and the role Cu/ZnSOD plays in ginseng and will create a foundation to develop a highly efficient production method of ginseng Cu/ZnSOD.

## 2. Results and Discussion

### 2.1. Cloning and Expression of pBV220-SOD

**Figure 1:**
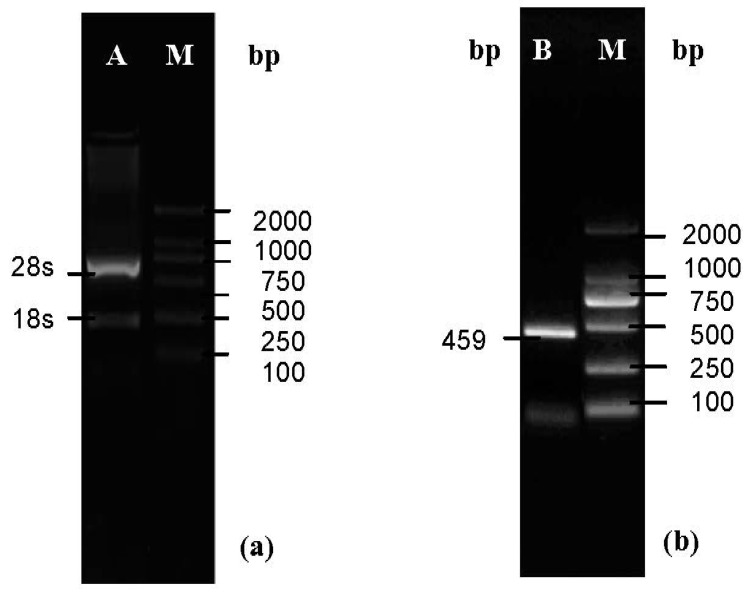
Nucleic acid electrophoresis analysis of the RNA and PCR products. (**a**) Line A, the total RNA of ginseng, 28S/18S was 2.5. Line M, DL2000 Marker; (**b**) Line B, RT-PCR products of ginseng Cu/ZnSOD, the 459 bp band was present. Line M, DL2000 Marker.

The quality of the total RNA extracted from ginseng was characterized by electrophoresis through 1% agarose gels. The integrity and purity of the RNA samples was confirmed when the 28S band was clear and bright and the 28S/18S ratio was more than 2 (Figure 1a).

The complete cDNA of SOD was amplified by RT-PCR. On the basis of the sequence of Cu/ZnSOD in Korean ginseng, gene specific primers were designed, and SmaI and SalI sites were included in the primers to facilitate the cloning of Cu/ZnSOD. The PCR product was characterized by agarose gel electrophoresis. The 459 bp band appeared as expected (Figure 1b).

**Figure 2:**
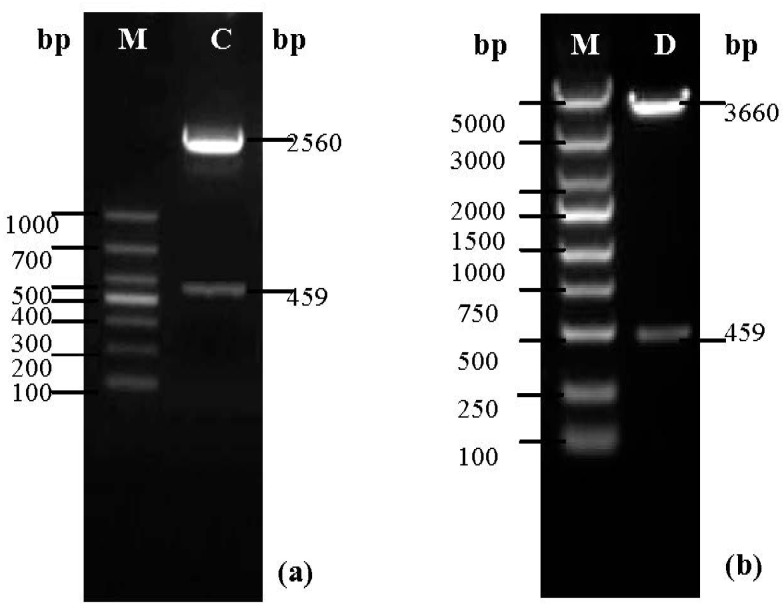
Nucleic acid electrophoresis analysis of the recombinant plasmids. (**a**) Line C, the bands of pMD18-T (2560 bp) and SOD (459 bp). Line M, DL2000 Marker; (**b**) Line D, the bands of pBV220 (3660 bp) and SOD (459 bp). Line M, DL5000 Marker.

The *E. coli* colonies containing the recombinant plasmid pMD18-T-SOD were amplified by PCR. A new plasmid was extracted from the PCR product and identified by double digestion analysis with Sma I and Sal I enzymes. The predicted bands of pMD18-T (2560 bp) and Cu/ZnSOD (459 bp) were visualized as shown by agarose gel electrophoresis (Figure 2a), indicating that the Cu/ZnSOD gene was successfully ligated into the pMD18-T vector.

In the same manner, after transformation, bacterial culture, PCR, plasmid extraction and double digestion, the recombinant plasmid pBV220-SOD was analyzed by agarose gel electrophoresis. Bands of sizes corresponding to pBV220 (3660 bp) and Cu/ZnSOD (459 bp) were visualized as shown in the figure below (Figure 2b).

When transformation was indicated by gel electrophoresis, it was confirmed by sequencing at BGI Co. Sequence analysis indicated that the target Cu/ZnSOD gene was 459 bp long (Table 1) and codes for 152 amino acid residues. The molecular weight of the protein predicted by the network program was 15.3 kDa [13]. 

**Table 1:** Sequence analysis of Cu/ZnSOD gene and its encoding amino acids.

	ATG GTG AAG GCT GTC ACA GTT CTT AGC GGC AGT GGA GGA GTC AGT GGC GTC ATC CAC TTT
**1**	**M V K A V T V L S G S G G V S G V I H F**
	ACC CAG GAA GAA GAT GGT CCA ACT ACA GTT ACT GGA AAA CTT TCT GGC CTT GCA CCC GGA
**21**	**T Q E E D G P T T V T G K L S G L A P G**
	CTT CAT GGT TTT CAT GTC CAT GCA CTT GGT GAT ACA ACA AAC GGT TGC CTG TCA ACT GGA
**41**	**L H G F H V H A L G D T T N G C L S T G**
	CCC CAT TAT AAC CCT GCT AAT AAA GAG CAT GGT GCT CCG GAA GAT GAG ACC CGC CAT GCT
**61**	**P H Y N P A N K E H G A P E D E T R H A**
	GGT GAT CTC GGG AAT GTG ACA GTT GGT GAA GAT GGT ACT GCC GAA TTC ACT ATT GTT GAC
**81**	**G D L G N V T V G E D G T A E F T I V D**
	AAA CAG ATT CCA CTC ATT GGA TCA GGT TCC ATC ATT GGA AGG GCC GTA GTT GTC CAT GCT
**101**	**K Q I P L I G S G S I I G R A V V V H A**
	GAC CCT GAT GAC TTG GGA AAG GGT GGT CAT GAA CTC AGC AAA AGC ACT GGA AAT GCT GGT
**121**	**D P D D L G K G G H E L S K S T G N A G**
	GGA AGG CTT GCC TGT GGT TTC ATT GGG CTG CAG GGT TGA
**141**	G R L A C G F I G L Q G *

* The full-length of the sequence was 459 bp and codes for 152 amino acid residues.

By comparing our ginseng SOD sequencing results with the results published in GenBank by Blast in NCBI, we found one base pair difference between the obtained sequence and the previously published sequence at the 439 site (“A” for the ginseng sequencing and “T” in GenBank). These results suggest that the ginseng Cu/ZnSOD gene is almost completely homologous with the published gene (99%).

### 2.2. Expression of the Recombinant Protein

**Figure 3:**
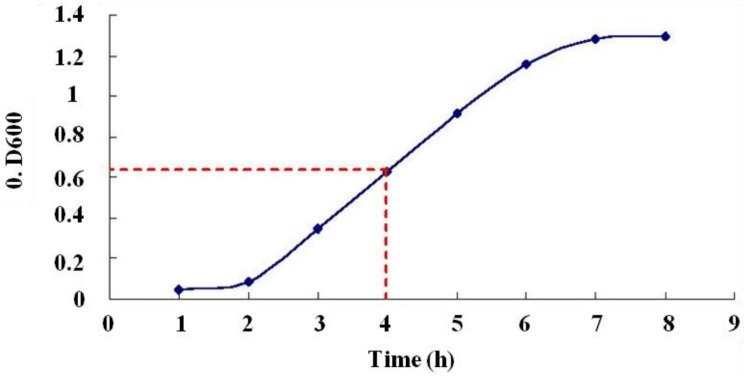
Growth curve of *E. coli* BL21(*DE3*) containing pBV220-Cu/ZnSOD plasmid. The OD 600 reached 0.6 at 4 h.

*E. coli* containing the pBV220-Cu/ZnSOD genes was cultivated in a shaker at 200 rpm and 30 °C, and the OD was measured every hour at 600 nm. The bacteria growth curve was the S-type, and expression of the plasmid was induced at 4 h when the bacteria were in the logarithmic growth phase (Figure 3).

**Figure 4:**
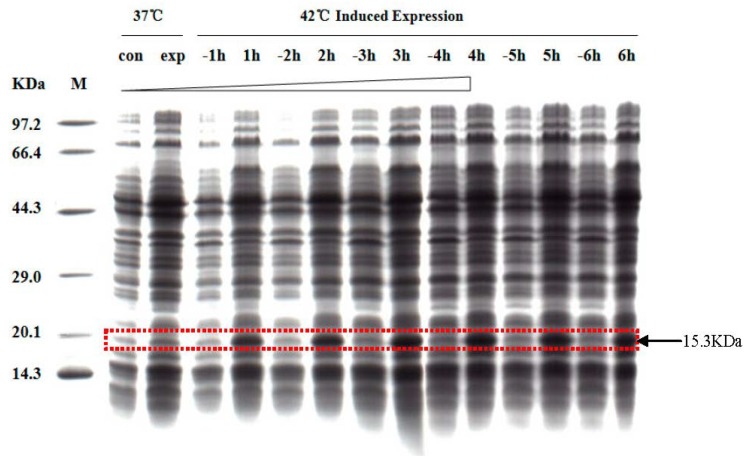
SDS–PAGE analysis of the effect of induction time for expressing bacteria and control bacteria. Line M: molecular weight marker: 97.2 kDa, 66.4 kDa, 44.3 kDa, 29.0 kDa, 20.1 kDa, and 14.3 kDa; con: control bacteria before induction; exp: expressing bacteria before induction; Lines −1 h, −2 h, −3 h, −4 h, −5 h, and −6 h: control bacteria induced for 1, 2, 3, 4, 5, and 6 h, respectively; Lines 1 h, 2 h, 3 h, 4 h, 5 h and 6 h : expressing bacteria induced for 1, 2, 3, 4, 5, and 6 h, respectively. The observed 15.3 kDa band on the *E.coli* BL21 (*DE3)* containing the pBV220-Cu/ZnSOD plasmid was much stronger than that of the control, and the greatest expression (9.94%) occurred at 4 h.

To identify the induced expression time of Cu/ZnSOD protein, the expressing bacteria and control bacteria were analyzed every hour by SDS-PAGE. The results confirmed that the predicted 15.3 kDa recombinant protein was present in the transformed but not the control bacteria. The time of the maximum amount of induced expression of the protein was 4 h, as expected (Figure 4).

### 2.3. Purification of the Recombinant Protein

**Figure 5:**
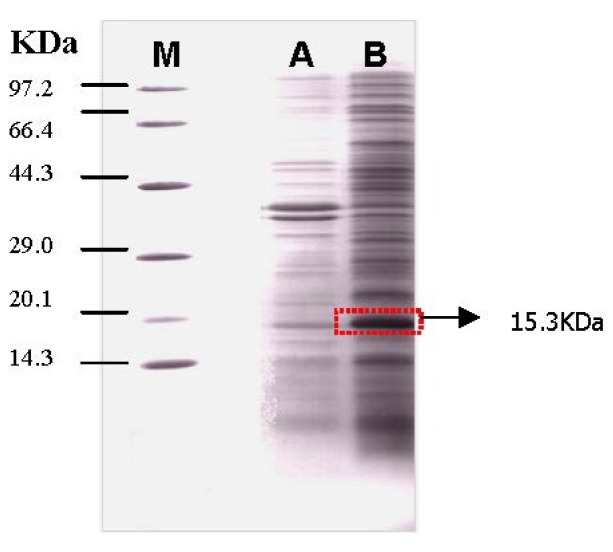
SDS–PAGE analysis of lysed bacteria. Line M: molecular weight marker (97.2 kDa, 66.4 kDa, 44.3 kDa, 29.0 kDa, 20.1 kDa, 14.3 kDa); Line A: supernatant of the lysed bacteria; Line B: precipitate of the lysed bacteria.

The expressing bacteria were lysed, and both of the soluble and insoluble fractions were analyzed by SDS-PAGE. The target band was present in the precipitate, indicating that the expressed protein was present as inclusion bodies (Figure 5).

**Figure 6:**
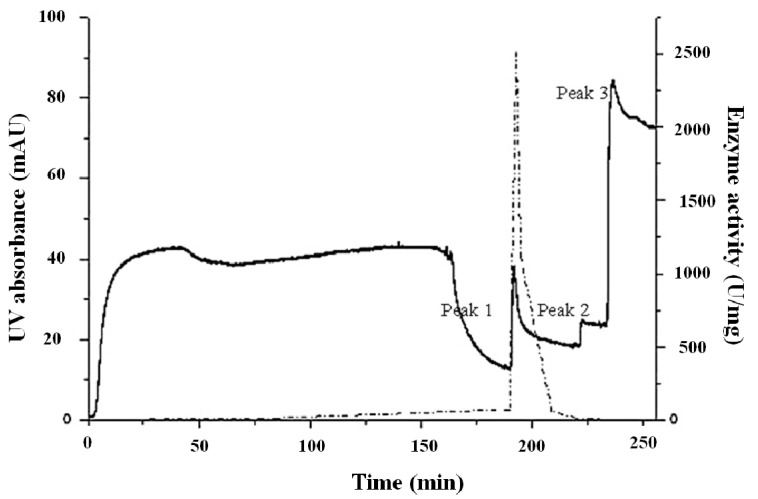
The chromatogram of SP-Sepharose and enzymatic assay fractions. The dotted line was the enzyme activity of the fractions. There were three peaks appeared with UV absorption. Peak 1 was the target protein, Cu/ZnSOD.

**Figure 7:**
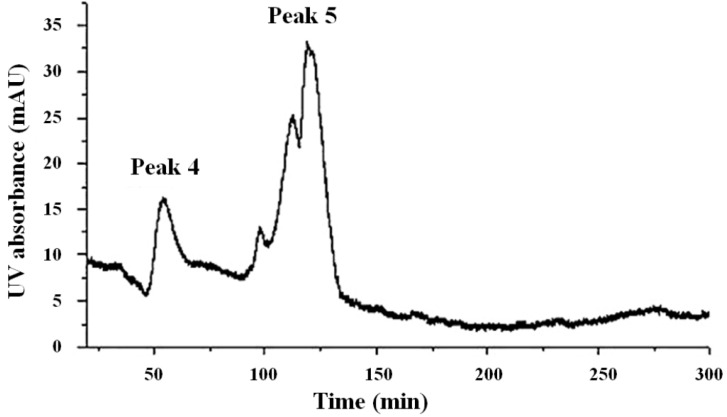
The chromatogram of Sephadex G-75. Peak 4 corresponds to Cu/ZnSOD.

**Figure 8:**
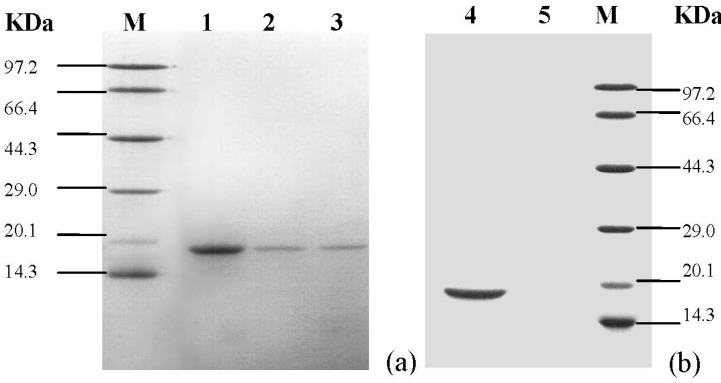
SDS–PAGE analysis of the purified protein. (**a**) SDS–PAGE of the 3 peaks eluted from the SP-Sepharose; line 1 corresponds to the crude Cu/ZnSOD; (**b**) SDS–PAGE of the of the 2 peaks eluted from the Sephadex G-75 column, line 4 corresponds to the pure Cu/ZnSOD.

The inclusion body solution was concentrated, purified by SP-Sepharose, and then eluted with NaCl. The chromatogram of the SP ion-exchange had three peaks (Figure 6). For the purpose of purification, the active fractions were purified by being passed through a Sephadex G-75 column at 280 nm (Figure 7). For the purified sample, a single band with an apparent molecular weight of approximately 15.3 kDa was observed on SDS–PAGE (Figure 8).

### 2.4. Enzyme Activity of Protein

The enzyme activities of each fraction of the SP-Sepharose elution were measured to establish an activity curve, and there was only one peak appeared, overlapped with SP-peak1 (Figure 6). The three products of the crude enzyme (crude SOD), the fraction of peak1 from the SP-Sepharose column (SP-SOD) and the pure protein (G75-SOD) were measured for enzyme activity (Table 2).

**Table 2:** Enzyme activity of Cu/ZnSOD products.

Purified Products	Protein Content (mg/mL)	Enzyme Activity (U/mL)	Enzyme Specific Activity (U/mL)
Crude SOD	3.055	1145.37	374.96
SP-SOD	0.574	2005.57	3488.17
G75-SOD	0.965	9061.3	9389.96

Following manufacturer’s protocol, SOD activity was assayed using the SOD Assay Kit (Njjcbio, Inc, Najing, China). One unit of enzyme activity was defined as the amount of enzyme in 1 mL reaction solution needed to exhibit 50% dismutation of the superoxide radical.

### 2.5. Stability Determination of the Purified Recombinant Cu/ZnSOD

**Figure 9:**
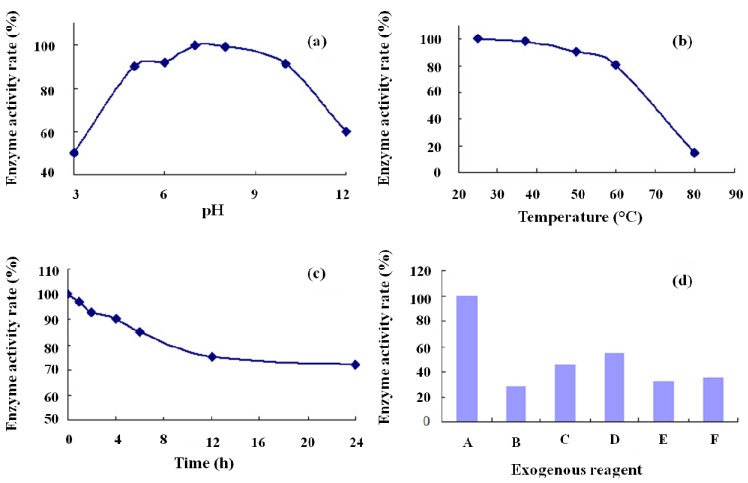
Characterization of recombinant Cu/ZnSOD. (**a**) The influence of pH (3, 5, 6, 7, 8, 10, and 12) on SOD activity was determined at 4 °C for 30 min; (**b**) The enzyme activity was measured at various temperatures (25, 37, 50, 60, and 80 °C); (**c**) The stable time was measured at 37 °C; (**d**) The influence of exogenous reagents (A: Control with no exogenous reagent, B: Added 67% methanol, C: Added 67% acetonitrile, D: Added 1 M guanidine hydrochloride, E: Added 3 M urea, and F: added 50 mM H_2_O_2_) on SOD activity.

The purified recombinant Cu/ZnSOD exhibited optimal activity at pH 5–10 (Figure 9a), but its activity decreased significantly when the solution was too acidic or too alkaline. The thermostability experiment demonstrated that the enzyme maintains high thermal stability (81.2% residual activity) after 30 min of incubation at 60 °C (Figure 9b). In addition, 83.7% of the enzymatic activity remained after incubation at 37 °C for 6 h and the decrease progressively tends to be stable at 12 h later (Figure 9c). The residual enzyme activity of Cu/ZnSOD when exposed to the exogenous agents tested was 28%–55% (Figure 9d).

## 3. Experimental

### 3.1. Plasmids, Strains and Media

*E. coli* DH5α (TakaRa, Dalian, China) was used as the host strain for cloning and maintaining the plasmid, and *E. coli* BL21 (*DE3*) (TakaRa) was used for protein expression. The strains were cultured in Luria-Bertani (LB) broth (1% tryptone, 0.5% yeast extract, and 1% sodium chloride) supplemented with ampicillin (50 μg/mL). For solid media, 2% agar was added. The cloning vector pMD18-T was purchased from TakaRa, and the expression vector pBV220 was obtained from the Changchun Institute of Biological Products. All restriction enzymes and the Expand High Fidelity PCR system were purchased from TakaRa. The sequences of the PCR products used for cloning were confirmed by sequencing at BGI Co., Ltd (Beijing, China).

### 3.2. RNA Isolation from Panax Ginseng

Following manufacturer’s protocol, total RNA was extracted from leaves of Panax ginseng using the TRIzol Kit (Invitrogen, California, Carlsbad, USA) [14,15]. The quality of the RNA was determined by agarose gel electrophoresis and gel imaging analysis.

### 3.3. Primer Design and Cloning of Cu/ZnSOD

The PCR primers were designed according to the multiple cloning sites of pBV220 and the Cu/ZnSOD sequence in the GenBank database under Accession No. AAB87572.1. The sequences are shown below; the upstream and downstream primers contain SmaI and SalI restriction sites, respectively:

Forward: 5'-
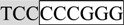
ATGGTGAAGGCTGTCACAG-3'


Reverse: 5'-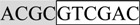
TCAACCCTGCAGCCCAATG-3'



The reverse transcription reaction was performed according to the TaKaRa RNA PCR Kit (AMV) Ver. 3.0 instructions. The PCR amplification of Cu/ZnSOD was performed as the following cycles: initial denaturation at 95 °C for 5 min; 30 consecutive cycles of denaturation at 95 °C for 45 s, annealing at 60 °C for 45 s, and extension at 72 °C for 50 s; and a final extension at 72 °C for 8 min. The amplification product was recovered and analyzed by electrophoresis on a 1% agarose gel stained with ethidium bromide.

### 3.4. Construction of pBV220-Cu/ZnSOD

The Cu/ZnSOD gene was ligated into the pMD18-T vector. Then, the pMD18-T-SOD plasmid was transformed into *E. coli* DH5α cells [16]. The transformants were grown on LB agar plates supplemented with ampicillin (AMP, 50 μg/mL) at 30 °C for 20 h. LB medium was inoculated with some white bacterial colonies, and the bacteria were cultivated at 200 rpm and 30 °C for 12 h. Then, the plasmids extracted from the bacterial liquid were digested with the Sma I and Sal I restriction enzymes and run on a 1% agarose gel.

The positive clones were selected, recovered and ligated into the pBV220 vector. *E. coli* BL21(*DE3*) was transformed with the recombinant plasmid construct and transformed colonies were selected for on LB agar plates containing 50 μg/mL Amp. The transformants were cultured at 30 °C in a shaker at 200 rpm for 12 h. Successful creation of the recombinant plasmid was confirmed by double digestion and electrophoresis on a 1% agarose gel, and positive clones were identified by sequence analysis. The correct recombinant expression vector was named pBV220-Cu/ZnSOD.

### 3.5. Recombinant Protein Expression

The recombinant protein was expressed in the *E. coli* strain BL21(*DE3*) and selected on LB agar plates containing 50 μg/mL Amp at 30 °C overnight. Liquid LB medium was inoculated with some white bacterial colonies, and the bacteria were cultivated in a shaker at 200 rpm and 30 °C for 12 h.

To determine the growth curve of the bacteria, it was sampled at 1, 2, 3, 4, 5, 6, 7 and 8 h. After inoculating 10 mL of LB medium with the bacterial samples at a ratio of 1:100, the OD of the bacteria was measured at 600 nm.

After inoculating 100 mL of LB medium with bacterial strains containing pBV220-SOD and control bacteria, the bacteria were cultivated in a shaker at 200 rpm and 30 °C for 12 h. When the OD of the bacteria at 600 nm reached approximately 0.6–0.8 with the temperature at 42 °C, protein expression was induced. To optimize the induced expression time, different sampling times were tested. One milliliter of bacterial broth was collected each hour and centrifuged at 10,000 rpm for 30 s before examining the precipitates by 10% Tris-glycine SDS-PAGE.

### 3.6. Inclusion Body Isolation

To examine whether the recombinant protein existed in the form of inclusion bodies, we chose the ultrasonic method and lysozyme bacteria method to lyse the bacteria. The cells were centrifuged at 8,000 rpm for 5 min and both of the supernatant and the precipitate were collected and analyzed by SDS-PAGE.

The precipitate obtained above and the cell lysates buffer containing 0.2 M NaOH and 1% SDS were mixed and centrifuged twice at 4 °C and 8,000 rpm for 5 min to collect the inclusion bodies. Then the inclusion body lysates buffer was added and stirred at RT for 1 h to help it dissolve, centrifuged at 4 °C and 8,000 rpm for 10 min. The supernatant was the crude enzyme. It was collected to prepare for the protein purification.

### 3.7. Protein Purification

The crude enzyme was dialyzed with 20 mM ammonium acetate buffer (pH 5.0) at 4 °C for 12 h and centrifuged at 10,000 rpm for 10 min. The supernatant was prepared, run through a 0.45 mm filter, and then applied to an SP-Sepharose Fast Flow column (1 × 10 cm) pre-equilibrated with ammonium acetate buffer. The column was washed with ammonium acetate buffer until no protein was detected. The adsorbed protein was eluted 3 times with increasing concentrations of NaCl (0 M, 0.2 M and 1 M) at a flow rate of 1 mL/min. Each resulting fraction was analyzed by SDS–PAGE, the Bradford method, and xanthine oxidase activity analysis.

The fractions with high SOD activity were lyophilized, dissolved with 1 mL of water, and then loaded onto a pre-equilibrated Sephadex G-75 column (1 × 100 cm). The proteins were eluted with water at a flow rate of 0.5 mL/min. The purity of the SOD sample was confirmed by SDS-PAGE, the Bradford method and enzyme activity analysis.

### 3.8. Biochemical Characterization of Cu/ZnSOD

To determine the enzyme stability of Cu/ZnSOD at different pH values, the enzyme was pre-incubated with different buffers in the pH range of 3 to 12 at 4 °C for 30 min, and the enzyme stability was determined when the samples returned to room temperature (RT). To determine the thermostability of Cu/ZnSOD, the enzyme was pre-incubated for 30 min at various temperatures from 25 to 80 °C, and residual enzyme activity was assessed under standard conditions. To determine the time period of stable enzyme activity, the enzyme was pre-incubated for different time periods ranging from 0 to 24 h in a water bath at 37 °C. To investigate the effects of various exogenous reagents on the recombinant Cu/ZnSOD enzyme, its enzymatic activity was measured in 67% methanol, 67% acetonitrile, 1 M guanidine hydrochloride, 3 M urea and 50 mM hydrogen peroxide.

## 4. Conclusions

In summary, we have successfully cloned the Cu/ZnSOD gene from *Panax* ginseng into the pBV220 expression vector. The expression and purification of SOD outlined here generated approximately 965 mg of highly purified protein per liter of broth with a high enzyme specific activity of 9,389.96 U/mg. This study demonstrates that the Cu/ZnSOD gene is expressed in the form of inclusion bodies in *E. coli*, and its homology with the report in GenBank was 99%. These results provide a theoretical and experimental foundation for further studies on the oxidation resistance mechanism of *Panax* ginseng and the establishment of a convenient and efficient Cu/ZnSOD production method. This will be helpful for the development and application of Cu/ZnSOD.
